# Is objectively measured exposure to built and natural environment associated with population-level cardiovascular disease mortality in Great Britain?

**DOI:** 10.1016/j.ssmph.2025.101875

**Published:** 2025-10-25

**Authors:** Laura Macdonald, Natalie Nicholls, Fiona Caryl, Jonathan R. Olsen, Daniela Fecht, Richard Mitchell

**Affiliations:** a MRC/CSO Social and Public Health Sciences Unit, University of Glasgow, UK; b Institute for Social Science Research, The University of Queensland, Australia; c Small Area Health Statistics Unit (SAHSU), MRC Centre for Environment and Health, Imperial College London, UK

**Keywords:** Cardiovascular disease, Mortality, Deprivation, Geographic information systems, Tree cover, Air pollution

## Abstract

**Background:**

Cardiovascular disease (CVD) causes one-third of global mortality, with modifiable risk factors such as unhealthy diet, sedentary behaviour, tobacco/alcohol use contributing to 80 % of CVD deaths. The built environment (BE) can influence CVD risk indirectly by shaping health behaviours and directly through environmental exposures like air pollution. While research has established connections between isolated environmental features and CVD, this study addresses significant research gaps in understanding how multiple BE characteristics influence CVD mortality across socioeconomic contexts, aiming to inform neighbourhood design to reduce both CVD and inequalities.

**Methods:**

We modelled, for small areas across GB, tree cover, air pollution, walkability, densities of health-detrimental amenities (‘bads’) (e.g. fast-food outlets) and health-promoting amenities (‘goods’) (e.g. gyms), and income deprivation. Generalised linear models were used to assess associations between small area features and (sex-stratified) age-standardised CVD mortality rates (i.e. ICD-10 codes I00–I99), controlling for deprivation, urban-rural, country, and local authority. Combined models (i.e. models mutually adjusted for all BE features) identified the unique contribution of each feature while accounting for those that ‘co-located’. Interaction analysis was performed to examine variations by income deprivation.

**Results:**

A slight increase in CVD mortality risk was associated with greater ‘goods’ densities (female mortality ratio (MR):1.005 (CIs:1.003–1.007), p < 0.001, male MR:1.005 (CIs:1.003–1.006), p < 0.001), and higher air pollution (female MR:1.006 (CIs:1.003–1.009), p < 0.001, male MR:1.008 (CIs:1.005–1.009), p < 0.001). A slight decrease in CVD mortality was associated with higher walkability for females (MR:0.996 (CIs:0.992–0.999), p = 0.034) and tree cover for males (MR:0.999 (CIs:0.998–0.999), p = 0.007). Higher air pollution levels and ‘bads’ were associated with higher male CVD mortality in deprived areas.

**Conclusion:**

Findings have clear policy implications, suggesting prioritisation of reductions in air pollution—particularly in deprived areas—while promoting walkability and tree cover to reduce health inequalities. Unexpected positive associations between ‘goods’ and mortality highlight that complex neighbourhood effects warrant further study.

## Highlights

• Built environment (BE) influences CVD risk via behaviours and exposures.
• Study models multiple BE features across small areas in Great Britain.
• Higher air pollution linked to increased CVD mortality in both sexes.
• Walkability (females) and tree cover (males) linked to lower CVD mortality.
• Air pollution and amenity ‘bads' raised male CVD mortality in deprived areas.

## 1 Introduction

Cardiovascular disease (CVD) causes 17.9 million deaths annually, accounting for one-third of global mortality (WHO, 2021). CVD encompasses diseases affecting the heart and blood vessels, including coronary disease, heart attacks, stroke, and hypertensive heart disease (British Heart Foundation, 2024). Its impact extends beyond individual health outcomes to societal costs through healthcare expenses, lost productivity, and premature mortality (Luengo-Fernandez et al., 2023). Those of lower socio-economic status (SES) face both higher disease prevalence and earlier mortality (Schultz et al., 2018).

Modifiable risk factors account for 80 % of CVD deaths globally; these primarily include lifestyle factors such as poor diet, physical inactivity, and substance use, which increase blood pressure, cholesterol, blood glucose, obesity, and kidney dysfunction (British Heart Foundation, 2024). However, the built environment (BE) - comprising human-made spaces including buildings, roads, paths, and green spaces - also influences CVD risk through multiple pathways (Lai et al., 2023; Liu et al., 2023). Some BE features affect health behaviours through the availability of food outlets, exercise facilities, and green spaces for example, whilst other environmental factors like air pollution can directly impact cardiovascular health by damaging blood vessels through pollutant absorption (British Heart Foundation, 2022). While individual factors remain the strongest predictors of cardiovascular risk (Bays et al., 2021), both the difficulty of achieving and sustaining change in those factors and the population-wide impact of environmental modifications has sparked growing interest in the BE's influence on CVD (Koohsari et al., 2020).

Research increasingly examines the relationships between neighbourhood characteristics and cardiovascular morbidity and mortality rates. Systematic reviews and meta-analyses showed strong evidence linking neighbourhood walkability with reduced CVD risk factors (obesity, hypertension, type II diabetes (T2D)) (Chandrabose et al., 2022), and connecting greenspace and tree cover with lower CVD mortality (Wolf et al., 2020). An Australian study found that increased tree cover consistently correlated with lower odds of T2D, hypertension, and CVD, while total greenspace showed weaker associations only with T2D and hypertension (Astell-Burt and Feng, 2020). Research also demonstrated links between higher CVD morbidity/mortality and both air pollution (Liu et al., 2023) and fast-food density (Meijer et al., 2023). While research on how other neighbourhood factors beyond walkability, greenspace, and air pollution influence CVD has been surprisingly limited, studies showed connections between key health behaviours and environmental features. For example, sports facility density correlated with higher exercise rates (Kärmeniemi et al., 2018), healthy food retail access with improved diet (Dixon et al., 2021), higher tobacco outlet density with increased smoking rates (Valiente et al., 2021), and greater alcohol outlet presence with increased alcohol consumption levels (Sherk et al., 2018).

However, interpreting these individual associations is complicated by a fundamental methodological challenge: BE features rarely exist in isolation but ‘co-locate’ within neighbourhoods, making it difficult to determine which specific characteristics drive health outcomes. Most studies examine BE features individually, failing to account for their spatial clustering and potentially confounding effects. Leading scholars emphasise the need to move beyond studying isolated features to examine how multiple BE elements affect risk (Koohsari et al., 2020). Disentangling the unique contributions of multiple co-located features is a key challenge in understanding BE impacts on CVD and designing effective interventions.

The geographical distribution of these BE factors varies by socio-economic status. In Glasgow, Scotland for example, deprived areas showed higher concentrations of 'environmental bads' such as alcohol, tobacco, and fast-food outlets (Macdonald et al., 2018). However, this pattern wasn't universal. New Zealand research found that while deprived areas had more 'bads', some also offered better access to 'goods', such as sports facilities and supermarkets (Marek et al., 2021). Compared to affluent areas, deprived neighbourhoods typically faced higher pollution levels (Barnes et al., 2019) and less greenspace (Schüle et al., 2019) but exhibited better walkability (Macdonald et al., 2016). Conceptualising BE factors as 'bads' or 'goods' provides a useful framework for understanding ‘health-harming’ and ‘health-benefiting environments’ - this terminology offers a clear way to communicate these concepts to policymakers. Crucially, evidence suggests that BE features like walkability (Sarkar et al., 2018) and greenspace (Rigolon et al., 2021) may offer stronger benefits in deprived areas, highlighting the potential for targeted neighbourhood interventions to both improve population health and reduce health inequalities.

Understanding how the BE influences CVD remains an important research gap if environment is to be a lever to reduce both rates of, and inequalities in, CVD. Our study addresses this critical methodological gap by examining how multiple BE characteristics influence CVD mortality across Great Britain, using joint modelling approaches to disentangle the independent contributions of co-located features**.** Our research questions: are area-level measures of tree cover, air pollution, walkability, and densities of amenity ‘goods’ (e.g., sports facilities) and amenity ‘bads’ (e.g., fast-food outlets) associated with CVD mortality rates for women and men in GB? Do these associations vary across levels of area-level income deprivation?

We hypothesised that air pollution and 'bads' would correlate with higher CVD mortality, while tree cover, walkability, and 'goods', would show protective effects. We expected these associations to be more pronounced in deprived areas, and anticipated sex differences in these relationships, given documented variations in how females and males interact with, and are affected by, their local environments.

## 2 Methods

We obtained publicly available, routinely collected data on local demographic and socioeconomic characteristics; built environment features; and mortality due to cardiovascular disease.

### 2.1 Local area population, deprivation and context

We measured local area population, deprivation and context using the common spatial framework of data zones (DZ) (n = 6976) for Scotland, and Lower Super Output Areas (LSOAs) for England and Wales (n = 32,844 and 1909 respectively). These are small area units created for reporting population statistics, including mortality. We obtained mid-2015 to mid-2019 population estimates for these small areas by 10-year age-group and sex (from National Records for Scotland (NRS) and Office for National Statistics (ONS)) (as CVD burden varied by age/sex (Roth et al., 2020)). To measure area-level income deprivation, we used DZ-level Scottish Index of Multiple Deprivation income ranks (2016), and LSOA-level English (2015) and Welsh (2014) Indices of Multiple Deprivation income ranks (income ranks divided into quintiles). These indices are widely used in public health and health inequalities research to capture relative socioeconomic disadvantage of areas (Health Equity Evidence Centre, 2024).

We used only the income domains from each nation's deprivation index rather than the composite indices - these domains measure similar concepts of financial disadvantage across the nations. We obtained the Government's urban/rural classifications which provide a consistent way of defining urban and rural areas (Gov UK, 2023). We included urban/rural classification as a key factor because the distribution and nature of environmental features (e.g., walkability, air pollution levels, access to amenities) vary between urban and rural settings (CMO, 2024).

### 2.2 Neighbourhood environment features

Neighbourhood environment features were divided into: tree cover, air pollution, walkability, amenity 'bads', and amenity 'goods' (see Table 1). Features were carefully selected based on both their established links to CVD and reliable availability across GB, focusing on features that are both conceptually relevant and measurable. Other dimensions (e.g., noise, housing quality, land use mix) were considered but not included due to limited data availability or correlation with selected features. Here, we briefly describe included features - see additional detailed information about the features in the supplementary section.

**Table 1:** Summary of neighbourhood features including pathways to cardiovascular health (see supplementary text for full definitions).

Feature	Data source	Variables	Pathway to cardiovascular health
**Tree cover** (i.e., satellite-derived deciduous and evergreen trees/shrubs).	European Union's (EU) Copernicus Land Monitoring Service (CLMS) (2018)	DZ/LSOA-level percentage tree cover	Health-beneficial - environment (land temperature), body and mind (exercise, stress reduction)
**Air pollution** (i.e. annual average NO_2_ (μg/m^3^) modelled using land cover, population density, roads, traffic, topography etc).	See (Wang et al., 2022), (2019)	Continuous numeric values	Health-detrimental – direct influence on disease risk
**Walkability** (Z scores measuring street connectivity and dwelling densities)	OS MasterMap Highways Network’ (Paths and Roads) data (2017), ONS/ORS dwelling densities (from 2011 Census)	Continuous numeric values	Health-beneficial - influence on exercise/active travel
**Bads** (i.e. (on- and off-sale) alcohol, tobacco, and fast-food outlets)	Ordnance Survey (OS) Points of Interest (POI) (2017)	Numbers of bads per 1500 population	Health-detrimental - influence on alcohol, smoking, diet/weight
**Goods** (i.e. ‘healthy’ food retail e.g. supermarkets, and sports and leisure facilities)	OS POI (2017)	Numbers of goods per 1500 population	Health-beneficial - influence on diet/weight and exercise

We measured DZ/LSOA-level tree cover percentage using satellite-derived data from the European Union's (EU) Copernicus Land Monitoring Service (CLMS), which combined forest and small woody features (for 2018) (CLMS, 2018a; CLMS, 2018b). Using R version 4.4.2 (R Core Team, 2024), we created a tree cover raster by combining datasets, extracted tree cell counts, and calculated percentage tree cover for each DZ/LSOA.

We assessed air pollution using existing, extensively validated, modelled annual average ambient nitrogen dioxide (NO_2_) concentrations for 2019, using data on land cover, population density, roads, traffic, topography etc (Wang et al., 2022). We used NO_2_ as an indicator of ambient air pollution as it is a well-established marker of traffic-related and combustion-source pollution, strongly implicated in cardiovascular risk (Huang et al., 2021). We averaged values across 500m grid cells within each DZ/LSOA. The unit of measurement was micrograms per cubic metre (μg/m^3^).

We quantified walkability through a composite measure combining street connectivity (intersection density) derived from 'OS MasterMap Highways Network' (Paths/Roads) data (2017) and dwelling density, standardised as z-scores for each DZ/LSOA. The following formula was used: *walkability score = (2 x intersection z-scores) + (dwelling density z-scores)*, with weighting consistent with previous established methodology (Frank et al., 2009).

Our ‘bads’ definition was based on previous work utilising local government data on alcohol, tobacco and fast-food licensed outlets (Macdonald et al., 2018), while our definition of ‘goods’ was based on previous work categorising amenities influencing diet and exercise e.g., ‘healthy’ food retail and sports facilities (Marek et al., 2021). To create 'bads' (e.g., alcohol, tobacco, and fast-food outlets) and 'goods' (e.g., health food stores and sports facilities), we obtained 'Ordnance Survey (OS) Points of Interest (POI)' (points) data (2017). We calculated densities of bads per 1500 population, and densities of goods per 1500 population, for each DZ/LSOA.

### 2.3 Heart disease mortality

We obtained mortality records from NRS and ONS vital events data for every year 2015–2019 by sex and age-groups for DZs/LSOAs. Pre-2020 data was chosen to remove the impact of the COVID-19 pandemic. CVD mortality was grouped into a single 5-year outcome (2015-19) as standard practice, to remove annual fluctuation. Deaths for males and for females (by age) were from ‘circulatory/cardiovascular disease’ (e.g., hypertensive disease, ischemic heart disease, pulmonary heart disease, cerebrovascular diseases (including stroke) etc) as defined by ICD-10 codes I00–I99. Using the mid-year population estimates for the same years, mortality rates were age-standardised to the European Standard Population (ESP), giving a CVD mortality rate per 100,000 people for each DZ/LSOA. Age-standardisation rates were produced using the mortality counts for GB.

### 2.4 Statistical analysis - generalised linear models

The outcome was standardised mortality rate per 100,000 population for all cardiovascular diseases for males and females. All analyses were conducted separately for males and females, as is standard practice due to sex variation in CVD epidemiology (Woodward, 2019), to identify potential sex differences in environmental associations with CVD mortality. The main independent variables of interest were neighbourhood features identified on CVD pathways (Table 1), and the sex-stratified models also controlled for income deprivation quintiles, urban-rural classification and nested geographies of LSOA/DZ within Local Authorities (LA) (i.e. governing bodies responsible for services within specific geographic areas) and country. Controlling for nested geographies prevents bias by recognising shared and socioeconomic conditions that may influence CVD outcomes. This ensures independence assumptions are met, avoiding misleading results (Hawkins, 2012). Generalised linear models (GLMs) with quasi-Poisson were used to handle overdispersion. We first examined linear associations between CVD mortality and each independent variable of interest in separate models. We then estimated a *combined* model including all independent variables simultaneously to assess their independent effects while accounting for shared variance. This combined model reflects ‘real-life’ exposure to multiple BE features rather than isolated exposures. This approach allowed us to determine the unique contribution of each variable beyond their individual associations and to address potential ‘co-location’ of BE features (i.e. BE features that occur together in the same areas).

We considered whether to model LA as a random or fixed effect and tested it both ways before settling on fixed effects. Exploratory analysis revealed that up to 82 % of air pollution variation was structurally determined by LA characteristics, which fundamentally violated the random effects assumption of independence between LA traits and pollution levels. Model diagnostics demonstrated that LA fixed effects produced normally distributed residuals when grouped by LA, in contrast to models without LA controls or treating LA as a random effect which exhibited systematic bias. Moreover, since our analysis encompassed every LA in GB, we had no need to generalise to a wider population eliminating the primary remaining justification for a random effects approach. This choice particularly affected the association between CVD and air pollution which was non-sensical when treating LA as a random effect and which, on further investigation, revealed a classic case of aggregation bias or Simpson's Paradox.

To examine effect moderation by socioeconomic status, we extended our combined models to include interaction terms between income quintile and each environmental exposure of interest. We fitted interaction terms for each environmental exposure in separate models, while maintaining all other covariates from the main model. This approach allowed us to assess moderation of each environmental factor, while controlling for all other factors. For variables showing significant interaction effects, we generated predicted values across the range of exposures for each income quintile using the ggeffects package (Lüdecke, 2018) and visualised these relationships using ggplot2 (Wickham, 2016). All analyses were conducted using R version 4.2.3 (R Core Team., 2023).

## 3 Results

### 3.1 Mortality rate descriptive statistics

Examination of cardiovascular mortality rates across GB revealed substantial geographic and socioeconomic variation (Table 2). Scotland showed substantially higher rates than England, while Wales had intermediate rates. Rates were higher in urban than rural areas. A socio-economic gradient was evident, with mortality rates in the most deprived quintile higher than in the least deprived quintile.

**Table 2:** Female and male age-standardised CVD mortality rates (ASMRs), by country, urban-rural and deprivation quintile (Q1 = most deprived).

		Female ASMRs				Male ASMRs			
		mean	median	25th	75th	mean	median	25th	75th
**CVD**	**England**	207.5	186.4	129.1	258.3	320.7	292.2	208.6	399.2
	**Scotland**	287.1	232.4	137.9	354.4	408.1	343.7	211.5	515.2
	**Wales**	225.5	209.8	150.2	283.9	354.1	332.7	245.3	438.8

	**Rural**	207.2	181.7	128.7	246.4	304.6	275.1	203.3	365.7
	**Urban**	224.7	195.7	131.7	277.8	343.8	307.2	213.0	429.4

	**Q1 (most deprived)**	280.1	252.5	179.2	342.2	435.2	401.7	291.2	532.2
	**Q2**	244.5	217.6	154.3	296.7	377.9	344.4	251.9	461.9
	**Q3**	221.7	193.5	137.6	264.7	328.7	297.0	220.9	396.1
	**Q4**	194.1	171.3	119.5	234.6	292.3	264.8	192.8	353.0
	**Q5 (least deprived)**	167.6	145.6	97.3	206.4	250.1	224.9	157.0	306.7

### 3.2 Associations between CVD mortality and environment

Sex-specific combined models revealed distinct patterns of environmental influences on cardiovascular mortality (Table 3). To enhance interpretability, coefficients for 10-unit increases in each environmental factor are presented rather than single-unit changes. All variables had variance inflation factors (VIFs) below 2.5 indicating no multicollinearity issues.

**Table 3:** Predicted female and male CVD mortality ratios (MR) associated with 10-unit increases in environmental exposures, adjusted for income deprivation quintile, urban–rural classification, local authority, and country.

Sex	Environmental variable	Estimate	SE	p-value	Mortality Ratio	MR 95 % CI
Females	Tree cover	−0.005	0.003	0.134	0.995	0.988-1.002
	Air pollution	0.058	0.014	0.000	1.060	1.031-1.090
	Walkability	−0.039	0.018	0.034	0.962	0.927-0.997
	Bads	−0.004	0.004	0.306	0.996	0.989-1.004
	Goods	0.048	0.010	0.000	1.049	1.028-1.071
Males	Tree cover	−0.007	0.003	0.007	0.993	0.987-0.998
	Air pollution	0.076	0.011	0.000	1.079	1.056-1.103
	Walkability	0.009	0.014	0.541	1.009	0.981-1.038
	Bads	0.003	0.003	0.371	1.003	0.997-1.008
	Goods	0.048	0.008	0.000	1.049	1.032-1.066

Air pollution showed the strongest (harmful) effects, with a 10 unit increase in NO_2_ associated with a 7.9 % increase in male mortality (95 % CI: 5.6–10.3 %) and a 6.0 % increase in female mortality (95 % CI: 3.1–9.0 %). Sex-specific protective factors emerged from the analysis. A 10 % increase in tree cover was associated with a 0.7 % reduction in mortality (95 % CI: 0.2–1.3 %) for males. For females, although the association was not statistically significant, the point estimate did suggest a potential protective effect (0.5 % reduction). Conversely, a 10-point increase in walkability corresponded to a 3.8 % reduction in mortality (95 % CI: 0.3–7.3 %) for females but was null for males.

Counterintuitively, increased density of 'goods' was associated with higher mortality for both sexes, with a 10-unit increase corresponding to a 4.9 % rise in mortality rates for both males (95 % CI: 3.2–6.6 %) and females (95 % CI: 2.8–7.1 %). However, the density of 'bads' facilities showed no statistically significant association with mortality for either sex.

**Fig. 1:**
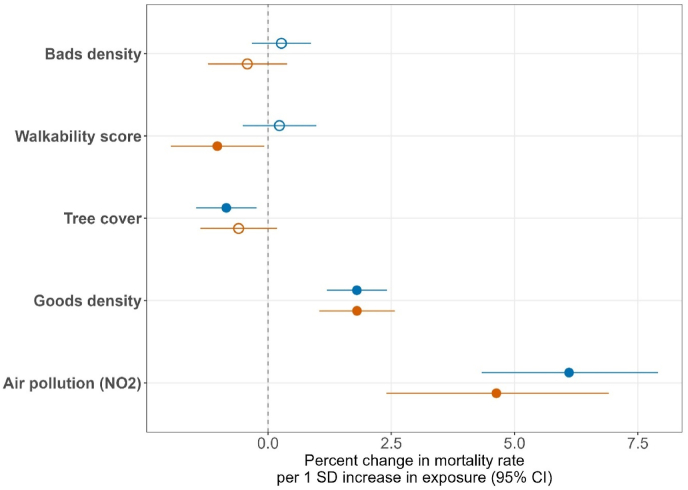
**Standardised effects of environmental factors on cardiovascular mortality by sex.**(Red = female, blue = male. Solid points indicate statistically significant effects. Effects are expressed as % change in mortality rate per 1 SD increase in each environmental factor.) (Note: Models adjusted for income deprivation quintile, urban–rural classification, LSOA/DZ nested within local authority, and country).

To directly compare the relative importance of different environmental factors, we standardised all continuous variables prior to refitting combined model (Fig. 1). This highlighted the substantially larger magnitude of air pollution effects compared to protective environmental factors, with the harmful effect of air pollution being approximately 7 times stronger than the protective effect of tree cover for men and 4 times stronger than the protective effect of walkability for women. Together, these findings suggest that while interventions targeting tree cover for men and walkability for women could offer sex-specific cardiovascular health benefits, reducing air pollution would likely yield the largest population health benefits for both sexes.

### 3.3 Effect moderation

**Fig. 2:**
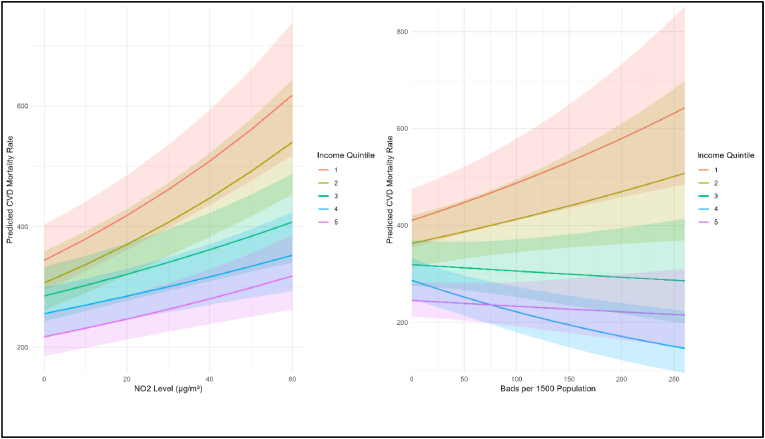
Male CVD mortality (ASMRs): income deprivation/air pollution (NO_2_ level) interaction and income deprivation/bads per 1500 population interaction (Q1 = most deprived). (Note: Interaction models adjusted for all covariates from the combined model (income deprivation quintile, urban–rural classification, LSOA/DZ nested within local authority, and country), with interaction terms fitted separately between income deprivation and each built environment exposure).

Interactions between deprivation and air pollution and between deprivation and bads were significant (both p < 0.001) among males only. Fig. 2 (left) illustrates interactions between deprivation and air pollution (NO_2_), and (right) between deprivation and bads densities in relation to male CVD mortality (i.e. predicted ASMRs).

All income groups showed increasing male CVD mortality as NO_2_ levels rise but a socioeconomic gradient is apparent—the most deprived quintile (Q1, red line) has consistently higher mortality rates than other quintiles. The steeper slope of the Q1 line as air pollution increased suggests that male deprived populations are more ‘at risk’ from the impacts of air pollution on CVD.

The socioeconomic pattern differs for density of ‘bads’. Whilst male CVD mortality increases with bads density in the most deprived quintiles (Q1 and Q2) the relationship changes in less deprived quintiles. Moderate deprivation (Q3) shows a relatively flat relationship. Whereas the least deprived quintiles (Q4 and Q5) showed decreasing CVD mortality as bads increase, that is, these groups appear ‘protected’ from the negative effects of increasing bads.

## 4 Discussion

### 4.1 Key findings

We hypothesised that air pollution and amenity ‘bads’ would be positively associated with CVD mortality, while tree cover, walkability and amenity 'goods', would show negative associations, and anticipated stronger associations in more deprived areas. Our analysis revealed significant associations, with some unexpected findings. While effect sizes were small, this is typical in ecological studies. Such area-level analyses of broader, more distal determinants often produce more subtle and dispersed relationships compared to individual-level studies examining more immediate causes (Macintyre and Ellaway, 2003). Air pollution was positively associated with CVD mortality of both sexes, while tree cover and walkability were negatively associated with male and female CVD mortality, respectively. Higher amenity 'goods' were surprisingly associated with slight mortality increases. Interactions between BE features and deprivation revealed significant differential impacts on male CVD mortality. Males in poorer areas appeared to demonstrate greater risk from air pollution and amenity ‘bads’. In contrast, males in less deprived areas showed reduced risk to air pollution effects and were somewhat protected against the negative impacts of increasing ‘bads’.

### 4.2 Comparison with other literature

**Tree cover -** As expected, we found a small but negative association between tree cover and CVD mortality for males only. Our findings are comparable with previous research, based in Australia, which found that for every 1 % increase in tree canopy, there were small reductions in the odds of prevalent type 2 diabetes (T2D) and CVD, as well as new cases of T2D, hypertension and CVD (Astell-Burt and Feng, 2020). Tree canopy coverage appeared to have more significant benefits to morbidity than total greenspace. A scoping review exploring research on urban trees impact on human health, reported that most of the studies provided evidence of urban trees’ positive influence on cardiovascular health (Wolf et al., 2020). Several mechanisms may explain the link between trees and CVD risk – trees may reduce health risks by moderating land temperature, promoting outdoor exercise, and fostering engagement with biodiversity enabling physiological restoration (Astell-Burt and Feng, 2020).

Our findings suggest gendered differences in treed space utilisation. While the association between tree cover and CVD mortality was statistically significant only for males, it's worth noting that the effect estimates for females showed a similar direction and magnitude (5 % decrease versus 7 % for males). This suggests the possibility of a modest protective association for women that may not have reached statistical significance due to other factors, such as, differential utilisation patterns of treed spaces by women. Women consistently report greater safety concerns in densely vegetated areas, with females experiencing greater levels of fear over crime within green space than their male counterparts (Maruthaveeran and van den Bosch, 2014). If women's safety perceptions of treed areas influence their physical activity patterns within these spaces, this could potentially contribute to differences in the magnitude of health benefits. Treed areas may feel unsafe to women due to inadequate lighting or maintenance, seclusion, or absence of others (Barker et al., 2025).

**Air pollution -** The finding of an association of NO_2_ with CVD mortality was expected as exposure to air pollution is known to increase CVD mortality risk through direct pathways. Direct effects include air pollution triggering systemic inflammation, blood vessel damage and narrowing, increased BP etc. Systematic reviews/meta-analyses found evidence of associations between NO_2_ and CVD mortality in short-/long-term exposure (de Bont et al., 2022). When exploring interactions between air pollution and income deprivation in associations with CVD mortality, we found a similar pattern to our 'bads' findings: males in deprived areas appeared to be more susceptible to the harmful effects of air pollution exposure. This sub-population may face multiple factors that create a concerning cumulative exposure burden. For example, they are more likely to smoke (ONS, 2023), causing a ‘synergistic effect’ or combined health impact that is more detrimental than the sum of individual exposures. Additionally, they are more likely to experience higher rates of pre-existing respiratory and cardiovascular conditions (ONS, 2022), which may be exacerbated by air pollution exposure (American Lung Association, 2023). These behavioural and health factors collectively reduce resilience to pollution-related cardiovascular stress - further compounded by deprived areas experiencing higher air pollution levels (Barnes et al., 2019).

**Walkability -** In agreement with our hypothesis, findings revealed a small but positive association between walkability and CVD mortality, but for females only. Existing literature reviews related walkability to factors influencing CVD. A systematic review and meta-analysis found strong evidence of longitudinal relationships between walkability and obesity, T2D, and hypertension - key intermediaries in CVD pathways (Chandrabose et al., 2019), however, few studies explored more direct associations with mortality (Lai et al., 2023; Nieuwenhuijsen, 2018). A UK-based study found that neighbourhood walkability was associated with lower BP and reduced hypertension risk, with particularly pronounced protective effects among females (Sarkar et al., 2018). The researchers suggested that walkability's stronger benefits for women may reflect sex differences in residential activity patterns, including time spent in the neighbourhood and the nature of activities performed.

Our findings similarly indicate sex-specific responses to walkability features. For women, safety appears to be a crucial determinant of walking behaviours (Golan et al., 2019). Safety may correlate with specific components of our walkability score, for instance, higher dwelling density creates greater street-level activity and enhanced perceived safety. Supporting this interpretation, one US study observed that proportionately more females utilised high-walkability streets compared to low-walkability streets, suggesting that more walkable designs on busier streets disproportionately attracted female pedestrians (Jensen et al., 2017). It's possible that our composite walkability score inadequately captures built environment factors that specifically influence men's walking behaviours. Further research is warranted to better understand which components of the built environment most effectively promote physical activity across different sex groups.

**Bads** - We found a significant interaction between densities of bads and area deprivation in relation to male CVD mortality, with opposing associations across the deprivation spectrum. In more deprived areas, higher densities of bads were associated with higher male mortality as hypothesised, while in less deprived areas, bads were associated with lower mortality. This contrasting pattern likely reflects the heterogeneous nature of retail outlets categorised as bads and their differential roles across socioeconomic contexts.

Men in deprived areas may face compounding disadvantage from both geographic and behavioural factors. Research shows men more frequently consume food prepared outside the home (including fast food) (Seguin et al., 2016) and have higher rates of tobacco and alcohol use compared to women (NHS England, 2024). In deprived neighbourhoods, bads may predominantly represent fast-food outlets and off-licenses that can amplify these existing behavioural tendencies through environmental cues and increased access. This environmental burden likely exacerbates existing health inequalities, as deprived populations already experience higher rates of chronic conditions and unhealthy lifestyle behaviours (Schultz et al., 2018). Conversely, in affluent areas, bads might in fact include higher-quality outlets, social clubs or supermarkets, with different health implications. This highlights the health impact of retail cannot be understood in isolation from the broader socioeconomic context in which it operates. Future research could explore more refined categorisation of bads to incorporate hierarchies of health-risk.

**Goods** - Unexpectedly, our findings revealed a positive association between goods and CVD mortality. Our findings are at odds with existing literature examining associations between goods and cardiometabolic risk. A New Zealand study reported that better access to ‘health-promoting’ BE features (such as sports facilities, greenspace, and fruit/vegetable stores), was related to reduced CVD risk factors, such as BMI and T2D (Hobbs et al., 2022). Similarly, a study exploring food and exercise environments, in the Netherlands, found that greater access to healthy food outlets and sports facilities, and greater walkability/bikeability, was associated with lower risk of higher BMI, BP, and hypertension (Lam et al., 2024).

Several contextual factors may explain this unexpected association. First, goods-rich areas may coincide with higher overall business density or mixed land use, which could be linked to both positive or negative health effects. Such environments may bring higher traffic, noise, or air pollution from commercial activity, potentially offsetting the benefits of healthy amenities. Second, socioeconomic processes may also play a role. A previous Scotland-based study found that access to goods was greater in the most deprived areas: perhaps more affluent/healthier population groups ‘self-select’ to areas with lower amenities density across all domains including 'goods' (Olsen et al., 2022).

Future research could test interactions between goods and other environmental factors to assess whether their health impacts depend on contextual conditions and to identify optimal configurations of built environment features. Although we used established categorisation of goods, further research could explore more refined and granular categorisation, for example, breaking down a broad category into more specific subcategories based on quality/variety of produce or services, or type. Previous Scotland-based research exploring associations between access to sports facilities and exercise frequency by facility type (i.e. public or private) found that greater access to private gyms only was related to increased exercise, potentially reflecting better facility ‘quality’ (Macdonald, 2019).

### 4.3 Strengths and limitations

Our research exhibited several strengths. We included various features of the neighbourhood BE, both health-beneficial or -damaging, based on robust spatial data, such as Ordnance Survey data - the most detailed and accurate mapping product available for the UK. We used comprehensive mortality and demographic data (NRS, 2020). A key strength of our research was using a combined model to determine the unique contribution of each BE feature beyond their individual associations and to address potential ‘co-location’ of features. We explored the role that deprivation played in associations between the BE and CVD, finding that males in deprived areas were more greatly vulnerable to bads – a finding key to health-related policy. Moreover, our methodology could be replicated in any nation with relevant mortality and spatial data.

We may be limited by our ecological design. While our analysis incorporated comprehensive mortality statistics for GB and accounted for age, sex, location, and deprivation, we lack individual-level data. Variables such as household income, ethnicity, neighbourhood tenure, smoking, healthcare access, and comorbidities could not be included, and the ecological nature of the study means causal pathways cannot be identified. Stronger causal inference would require longitudinal or cohort data (e.g., health records or panel surveys, such as, the UK Household Longitudinal Study) to capture individual-level exposures and outcomes over time. Nonetheless, our study provides valuable population-level insights.

While our modelling approach disentangles independent effects, it may not capture instances where combinations of environmental factors amplify or attenuate each other's health impacts. Future research could explore ‘cumulative risk’ approaches to examine potential synergistic effects between co-occurring exposures. Such approaches could provide a more comprehensive understanding of how real-world environmental exposure patterns influence health, although they do require careful consideration of multiple testing issues. Furthermore, while our approach addressed unmeasured geographic confounding within administrative boundaries, it may not fully capture the spatial clustering patterns inherent in environmental exposures and health outcomes. Unaccounted spatial autocorrelation could affect the precision of effect estimates. Here we establish a joint modelling framework for multiple BE features, and future studies could build upon this foundation by incorporating explicit spatial modelling to account for spatial autocorrelation in model residuals.

While our research captures spatial access to neighbourhood environment features, we acknowledge this is only one dimension of accessibility. We do not measure use patterns, or other barriers to use, such as, financial constraints (e.g., inability to pay costs for sports facilities or healthier food). However, spatial proximity is a necessary, though not sufficient, condition for potential health benefits. Finally, we understand our current conceptualisations of 'goods' and 'bads' - despite being grounded in established theory - risk oversimplifying the multidimensional pathways through which the BE interacts with health.

### 4.4 Policy implications

Our research has implications for policy. Much of the variation in mortality found here was minimal, however, the cumulative effect of multiple factors, could still be meaningful from a public health perspective. The observed associations underscore the need for individual-level investigations into complex relationships between environment and CVD, and policy formation that accounts for both socio-economic and gendered risk in environmental exposure.

Our standardised analysis underscores the disproportionate impact of air pollution, revealing that its harmful effect on cardiovascular mortality is approximately seven times stronger for men and four times stronger for women than the protective effects of tree cover and walkability, respectively. This substantial difference in effect magnitude suggests that air quality improvements should be prioritised in public health interventions aimed at reducing cardiovascular mortality.

While only 1.3 % of LSOAs exceed the previous WHO (2005) air quality recommended limit of 40 μg/m^3^ (WHO, 2021) (implemented in the UK under Air Quality Standards Regulations 2010), we observed that these areas are disproportionately concentrated in deprived areas, with 59 % of high-pollution areas falling within the two most deprived quintiles. Our analysis further revealed significant moderation of NO_2_ effects by area deprivation, with stronger associations between air pollution and cardiovascular mortality in the most deprived areas. Importantly, the associations we observed were not confined to LSOAs exceeding the 40 μg/m^3^ threshold, suggesting that even exposures well below the previous WHO limit may contribute to elevated CVD mortality. This aligns with growing evidence that there is no safe level of exposure to NO_2_ (Marks, 2022), and that incremental reductions in ambient concentrations—across the full exposure distribution—could yield meaningful population health benefits.

Based on our models, we estimate that implementing 2021 WHO air quality guideline of limiting NO2 to 10 μg/m^3^ would result in a 6.4 % overall reduction in male mortality. However, this effect varies substantially by area deprivation level—we estimate a 10.7 % reduction in mortality for males in the most deprived quintile compared to only a 4.1 % reduction in the least deprived quintile. For females, where no significant interaction was found, we estimate a consistent 4.9 % reduction across all deprivation levels. This gradient demonstrates how air quality interventions could simultaneously address both public health and health inequality concerns, with potentially more than twice the benefit in the most disadvantaged communities compared to the most affluent areas.

Our research critically challenges the key assumptions of 20-minute neighbourhood and local living policies, revealing that increasing 'goods' amenities does not automatically translate to improved health outcomes. Policy-led interventions should focus on urban planning to increase trees - tree planting is regarded as an economically viable solution that tackles both ecological challenges and population health concerns (Wolf et al., 2020). However, policymakers and planners must prioritise women's safety in the design and management of parks and wooded areas. Health equity requires targeted policies to address concentrated environmental hazards in deprived areas, yet current urban planning and public health legislation often fails to reduce socioeconomic inequalities in bads (Macdonald et al., 2018), or air pollution (Barnes et al., 2019; Gray et al., 2023), exposure. A more equitable approach would integrate environmental justice into planning decisions, and targeted investments and enforced environmental standards with consideration of socioeconomic disparities. This should include ensuring procedural justice through meaningful community participation, particularly in more deprived areas. Giving communities a voice in decision-making, ensuring transparent and impartial processes, treating residents with dignity and respect, and demonstrating trustworthy motives could strengthen the legitimacy of interventions, and improve the long-term sustainability of urban investments.

### 4.5 Conclusion

We examined associations of area-level measures of tree cover, air pollution, walkability, amenity 'bads', and 'goods', with population-level CVD mortality, for females and for males, and explored potential variation in associations by deprivation. Methodologically, this study makes an important contribution by using combined models to disentangle the unique effects of co-located built environment features - an approach that addresses a key limitation in environmental health research. This joint modelling approach allowed us to identify the independent contribution of each environmental factor while accounting for co-occurrence.

Our analyses revealed that tree cover (for males) and walkability (for females) were negatively associated with CVD mortality, while air pollution was strongly positively associated for both sexes. Importantly, our standardised analysis showed that the harmful effects of air pollution were approximately seven times stronger for men and four times stronger for women than the protective effects of tree cover and walkability. Furthermore, we found significant socioeconomic gradients in both exposure and vulnerability to air pollution, with males in the most deprived areas experiencing more than twice the mortality benefit from improved air quality compared to those in the least deprived areas.

These findings highlight the critical importance of addressing both deprivation and environmental factors to improve health equity. Our findings offer clear implications for future policy, suggesting that place-based planning should prioritise reducing air pollution - particularly in deprived areas - while also promoting walkability and tree cover, to simultaneously improve overall population health and reduce health inequalities.

## CRediT authorship contribution statement

**Laura Macdonald:** Writing – review & editing, Writing – original draft, Visualization, Validation, Software, Project administration, Methodology, Investigation, Data curation, Conceptualization. **Natalie Nicholls:** Writing – review & editing, Software, Methodology, Investigation, Formal analysis, Conceptualization. **Fiona Caryl:** Writing – review & editing, Software, Methodology, Investigation, Formal analysis, Conceptualization. **Jonathan R. Olsen:** Writing – review & editing, Methodology, Conceptualization. **Daniela Fecht:** Writing – review & editing, Methodology, Data curation. **Richard Mitchell:** Writing – review & editing, Methodology, Funding acquisition, Conceptualization.

## Ethics approval

This work did not require ethical approval as it used aggregated secondary data.

## Declaration of generative AI in scientific writing

The preparation of this work, authors used Claude 3.7 Sonnet to improve manuscript grammar, readability and language. After using this tool, the authors reviewed and edited the content and take full responsibility for the content.

## Funding

Laura Macdonald, Natalie Nicholls, Fiona Caryl, Jonathan Olsen, and Richard Mitchell were employed by the MRC/CSO Social and Public Health Sciences Unit, University of Glasgow, and supported by the Medical Research Council [grant number MC_UU_00022/4] and Chief Scientist Office [grant number SPHSU19]. Daniela Fecht acknowledges support from the MRC Centre for Environment and Health, Imperial College London, currently funded by the MRC (MR/S019669/1, 2019–2024).

## Declaration of competing interest

None.

## Data Availability

The authors do not have permission to share data.
