# Supplementary material for: Is objectively measured exposure to built and natural environment associated with population-level cardiovascular disease mortality in Great Britain?
Source: SSM Popul Health. 2025 Oct 25;32:101875. doi: 10.1016/j.ssmph.2025.101875 (PMC12615347; doi:10.1016/j.ssmph.2025.101875)
Supplement: Multimedia component 1 [file mmc1.docx]

**Supplementary text: Additional information about the built environment features**

**Tree cover** was assessed using satellite-derived data from the European Union's Copernicus Land Monitoring Service (for 2018). We used satellite-derived data as it provides a consistently measured, and geographically comprehensive, assessment of urban greenery across an entire region. We used three datasets at 10m resolution: 'Forest Type' - classifies forest cover as broadleaved, coniferous, or mixed, 'Forest Additional Support Layer' - identifies forest cover in agricultural and urban contexts, and 'Small Woody Features' - captures non-forest tree cover like hedgerows and bushes (CLMS, 2018a, CLMS, 2018b). Using R version 4.4.2 (R Core Team, 2024), we reprojected input rasters to British National Grid (BNG) coordinate system, created a binary tree cover raster by combining the datasets, extracted tree cell counts and calculated percentage tree cover for each DZ/LSOA.

**‘Air pollution’** - For air pollution we used existing, extensively validated, modelled average ambient nitrogen dioxide (NO^2^) concentrations, for 2019 (in micrograms per cubic metre (μg/m^3^)). Concentrations were modelled using spatial predictors including land cover, population density, road network, traffic, topography and buildings, and chemical transport. Further detailed information available (Wang et al., 2022). Air pollution (500m by 500m) grid cells were overlaid by DZ/LSOA boundaries and where grid cells intersected a DZ/LSOA their values were attributed to that DZ/LSOA. Mean NO^2^ values calculated for each DZ/LSOA.

**‘Walkability’** (i.e., densely populated, highly connected streets/paths). Walkability scores were derived from street connectivity and dwelling densities. Higher street connectivity indicates a greater number of route options for pedestrians, while higher dwelling densities indicates a greater number of nearby walking destinations (e.g., shops, public transport etc). Using GIS, street/path connectivity (for each DZ/LSOA) was calculated as intersection density, i.e., the ratio of true intersections (three or more connecting roads/paths) to the DZ/LSOA area. Dwellings per hectare at data/LSOA-level were obtained from NRS/ONS. To standardise measures and make them comparable, we converted both intersection density and dwelling density to z-scores (using IBM SPSS Statistics V.28). The following formula was used: *WS = (2 x intersection z-scores) + (dwelling density z-scores)* (weighting consistent with previous methodology (Frank et al., 2009)).

**Amenity ‘bads’ and ‘goods’.** Our definition of ‘bads’ was based on previous work utilising local government data on alcohol, tobacco and fast-food licensed outlets (Macdonald et al., 2018). Equivalent GB-wide data is unavailable, as an alternative we used relevant OS POI categories (i.e., outlets selling alcohol and/or tobacco - off-licenses, supermarkets, convenience stores, tobacconists, petrol stations, pubs, nightclubs, adult venues, social clubs, restaurants, hotels/inns), (outlets selling fast-food – fast-food outlets, and fish and chip shops). Our definition of ‘goods’ was based on previous work categorising amenities influencing diet and exercise e.g., ‘healthy’ food retail and sports facilities (Marek et al., 2021) (i.e., supermarkets, fishmongers, greengrocers, health food retail), (sports facilities athletics, bowling, climbing, and tennis facilities, golf courses, gymnasiums, sports halls, leisure centres, ice rinks, ski centres, sports grounds/pitches, squash courts, swimming pools, velodromes). Supermarkets were included in both bads and goods. Within ArcGIS Pro (version 3.1.3) co-ordinates of bads and goods (as points) were geographically located and overlaid with DZ/LSOA boundaries. Where a point fell within a DZ/LSOA polygon it was attributed this DZ/LSOA. Each DZ/LSOA was assigned a count of 'bads' and a count of 'goods', and densities were calculated per 1,500 population for each.
